# Ex vivo ultra-high field magnetic resonance imaging of human epileptogenic specimens from primarily the temporal lobe: A systematic review

**DOI:** 10.1007/s00234-024-03474-0

**Published:** 2025-03-08

**Authors:** Marie-Julie D. K. Lemmens, R. H. G. J. van Lanen, D. Uher, A. J. Colon, M. C. Hoeberigs, G. Hoogland, A. Roebroeck, D. Ivanov, B. A. Poser, R. P. W. Rouhl, P. A. M. Hofman, I. Gijselhart, G. S. Drenthen, J. F. A. Jansen, W. H. Backes, K. Rijkers, O. E. M. G. Schijns

**Affiliations:** 1 Department of Neurosurgery, Maastricht University Medical Center, Maastricht, The Netherlands; 2 Department of Radiology and Nuclear Medicine, Maastricht University Medical Center, PO box 5800, Maastricht, AZ 6202 The Netherlands; 3 Mental Health and Neuroscience (MHeNs) Research Institute, Maastricht University, Maastricht, The Netherlands; 4 Cardiovascular Research Institute Maastricht (CARIM), Maastricht University, Maastricht, The Netherlands; 5 Academic Center for Epileptology, Kempenhaeghe/Maastricht University Medical Center, Heeze/Maastricht, The Netherlands; 6 Centre d’Etude et de Traitement de l’Epilepsie, Centre Hospitalier Universitaire Martinique, Fort-de-France, France; 7 Department of Cognitive Neuroscience, Faculty of Psychology and Neuroscience, Maastricht University, Maastricht, The Netherlands; 8 Department of Neurology, Maastricht University Medical Center, Maastricht, The Netherlands; 9 Care and Public Health Research Institute (CAPHRI), Maastricht University, Maastricht, The Netherlands; 10 Department of Electrical Engineering, Eindhoven University of Technology, Eindhoven, The Netherlands

**Keywords:** Epilepsy, Epilepsy surgery, Ex vivo imaging, Ultra-high field, UHF MRI

## Abstract

**Purpose:**

Magnetic resonance imaging (MRI) is the preferred diagnostic tool for the detection of structural cerebral lesions in patients with epilepsy. Ultra-high field (UHF) MRI with field strengths ≥7 Tesla has been reported to improve the visualization and delineation of epileptogenic lesions. The use of ex vivo UHF MRI may expand our knowledge on the detection and detailed micromorphology of subtle epileptogenic lesions by bridging the gap between in vivo MRI and histopathology.

**Methods:**

A systematic review of available literature was conducted following PRISMA guidelines. A descriptive analysis of included articles was performed, focusing on (I) the ability of ex vivo UHF MRI to detect subtle abnormalities related to epilepsy, (II) different post-processing methods, and (III) concordance between UHF MRI and histopathology.

**Results:**

Eleven studies with focus on the depiction of focal cortical dysplasia (*n* = 4) or hippocampal sclerosis (*n* = 7) as causative lesion of drug-resistant epilepsy were included. Ex vivo UHF MRI proved its ability to visualize the anatomy of cortical and hippocampal structures in greater detail when compared to ex vivo conventional field strengths. Different MRI post-processing methods enabled differentiation between lesional subtypes and provided novel insights into (peri)lesional characteristics. Concordance between ex vivo UHF MRI findings and histopathology was high.

**Conclusion:**

Acquisition of ex vivo UHF MRI and its image processing has the potential to depict epileptogenic abnormalities in greater detail with a spatial resolution approximating histological images. The translation of ex vivo UHF MRI features to in vivo clinical settings remains challenging and urges further exploration.

**Supplementary Information:**

The online version contains supplementary material available at 10.1007/s00234-024-03474-0.

## Introduction

Epilepsy is the fourth most common chronic neurological disease, affecting approximately 68 million people worldwide [1, 2]. The majority of patients are effectively treated with antiseizure medication. However, 30–40% of patients do not reach acceptable seizure control and are classified as drug-resistant. A subgroup of patients with drug-resistant epilepsy (DRE) is eligible for resective brain surgery, which is an evidence-based curative treatment option [3–5].

The current initially recommended imaging method for detecting structural epileptogenic lesions in patients with epilepsy is 3 Tesla (T) magnetic resonance imaging (MRI) [6]. An “epileptogenic lesion-positive” MRI is a major predictor for the chance of postoperative seizure freedom [7]. However, despite recent advances in MRI technology, 20–30% of patients with focal epilepsy do not show epileptogenic abnormalities on brain MRI [8]. Beside the application of a dedicated epilepsy imaging protocol and evaluation by an epilepsy-trained neuroradiologist, the detection of these lesions also greatly depends on several technical factors, such as MRI field strength, the use of phased array head coils, and image processing and analysis. Neuropathological assessment remarkably reveals a focal lesion in 30–50% of MRI-negative patients. In these cases, focal cortical dysplasia (FCD) and hippocampal sclerosis (HS) are the most frequent findings [9–11].

Ultra-high field (UHF) MRI, with field strengths of 7T or higher instead of conventional 1.5T or 3T, stands at the forefront of neuroimaging techniques and may improve epileptogenic lesion detection [12]. To expand our knowledge on the characteristics of these lesions further, ex vivo imaging may be utilized as it provides several complementary benefits to in vivo scanning. More specifically, it is less constrained by acquisition time, permits easier repeat scanning of the same sample, is not affected by movement or vascular pulsatility, can use tighter fitting coils, and can correlate imaging findings in greater detail with histopathology [13–17].

Ex vivo UHF MRI of specimens derived from epilepsy surgery could potentially bridge the knowledge gap between in vivo clinical scanning and histopathology, especially in MRI-negative patients. Any new radiological biomarker in epilepsy could ameliorate the diagnostic process by improving identification, delineation, and classification of cerebral lesions associated with epilepsy [18–20]. Alongside the application of higher magnetic field strengths, other emerging MRI technologies, such as multi-coil designs and innovative post-processing methods, could provide additional information in ex vivo imaging [20–23].

Here, we systematically collect and review the literature on the use of ex vivo UHF MRI in human epilepsy surgery specimens. A unifying summary of available ex vivo UHF MRI data of epilepsy surgery specimens is necessary to select imaging characteristics which could be useful for in vivo clinical imaging of epilepsy patients and to reveal areas where further research is needed. For this study, we particularly focused on (I) the ability of ex vivo UHF MRI to identify subtle structural abnormalities related to epilepsy, (II) post-processing methods, and (III) concordance between ex vivo UHF MRI and histopathology.

## Methods

### Research protocol

This systematic review was written in accordance with the Preferred Reporting Items for Systematic Review and Meta-analysis Protocols (PRISMA) guidelines [24]. The review protocol consisted of a search strategy and in- and exclusion criteria for screening titles, abstracts, and full-text articles, as described below.

### Search strategy

The search strategy was kept as broad as possible, based on the search terms ‘epilepsy’, ‘UHF MRI’, ‘7T’ and ‘9.4T’. Utilized databases were PubMed, Cochrane Library, Embase (Ovid), Web of Science Core Collection, and ClinicalTrials.gov. Search queries were optimized for each specific database. We aimed at maximum sensitivity and kept search terms as broad as possible by also adding “free terms” alongside “MeSH terms” with the use of the Boolean logic operators “AND” and “OR”. References of collected papers were cross-checked manually to supplement the search. Since UHF MRI is a relatively recent development, the publication date did not need to be limited. The final search was performed on 26 September 2023. Full search strategies for each database can be found under supplemental information.

### Eligibility and study selection

Two authors (ML and RvL) independently screened titles and abstracts of retrieved articles based on the presence of search terms. After screening the database search results, full-text assessment was performed. Discrepancies regarding eligibility of studies were resolved through consensus and additional assessment by a third author (OS). The objective was to identify all published ex vivo UHF MRI studies investigating human brain tissue obtained after epilepsy surgery. Articles were included based on: specimens derived from epilepsy patients, MR images obtained at 7T or higher, ex vivo studies, original articles (i.e. no review paper), and publication in English language. Exclusion criteria were: non-human and in vivo studies, non-structural MRI studies, and no full text available.

### Quality assessment

Quality and risk of bias assessment of the included studies was performed independently by two authors (ML and RvL). The scoring tool of Oxford Centre for Evidence-based Medicine 2011 was used to determine the level of evidence [25]. Studies were rated on a scale of one to five, reflecting their study design, with systematic reviews ranked at level one and expert opinions at level five. Risk of bias was assessed using the NIH quality assessment tool for observational cohort studies and tool for case series [26]. Based on the quality assessment tool, the quality of studies was classified as good, fair, or poor.

### Study results

Included studies were ranked chronologically. Extracted data from each study included: study characteristics (authors, title, publication year, number of samples, study design), MRI parameters, histology features, inclusion and exclusion criteria, endpoints, outcomes, discussion remarks, and conclusion.

Pathological diagnosis was classified as HS (including mesial temporal sclerosis) and FCD. The current International League Against Epilepsy (ILAE) classification for the differentiation between FCD subtypes or HS subtypes was used [27, 28]. In case a different classification was used, cases were reclassified by two authors (ML and RvL) to their respective ILAE class based on the information available in the article. If the surgical procedure to obtain a hippocampal specimen was not specified, the resection was described as hippocampectomy.

## Results

### Selection of reviewed studies

**Fig. 1:**
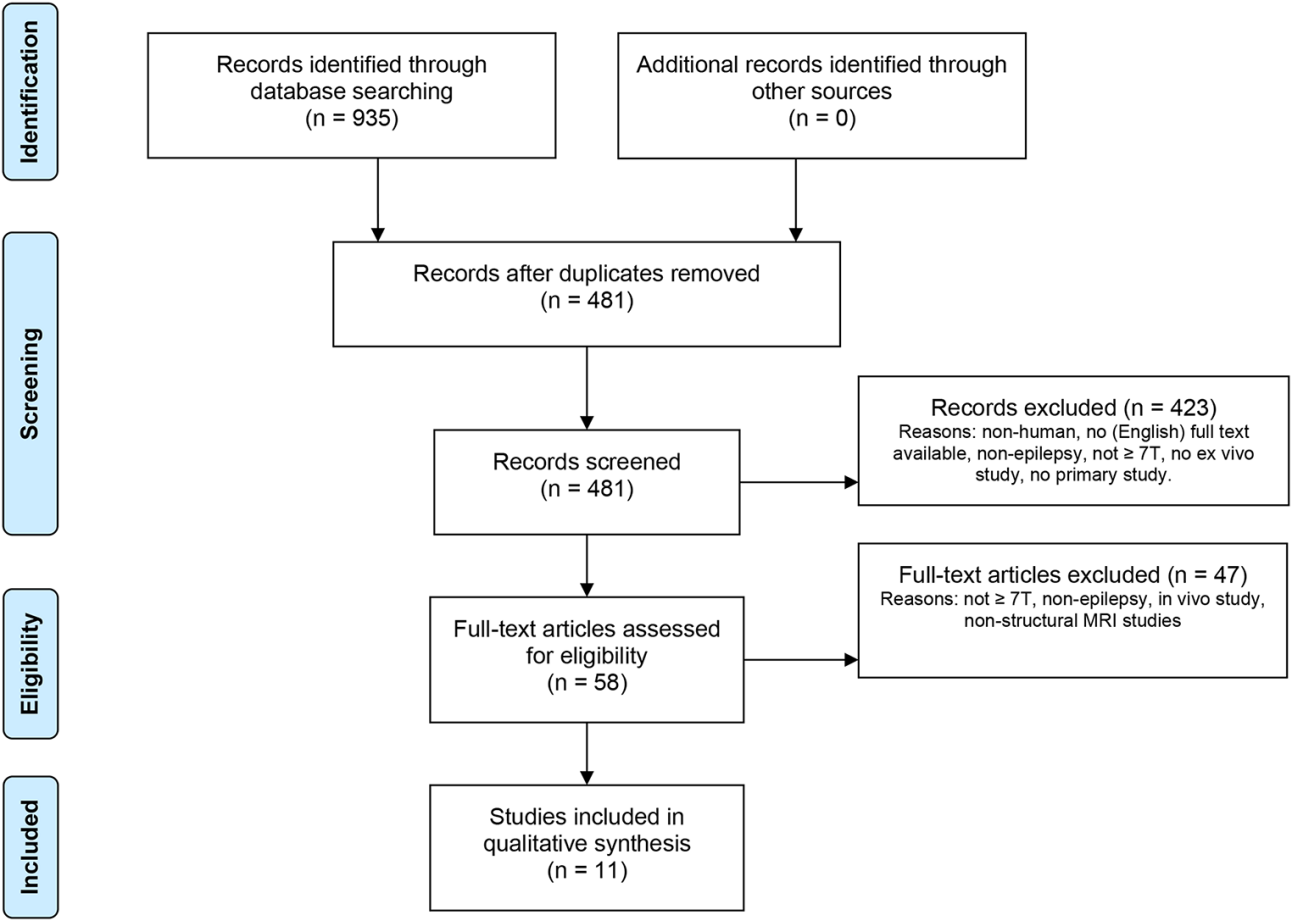
Flow chart of the article search, adapted after the PRISMA guidelines

Figure 1 shows the literature search and study selection process. The initial database literature search yielded 935 records. Eleven articles met eligibility criteria and were included in the qualitative synthesis.

### Study characteristics

Table 1 shows study design parameters of the included studies. Eight were prospective descriptive cohort studies, one retrospective, one case series, and one case report. Studies whose prospective or retrospective design was not specified in the full-text article were assessed independently by the reviewers, who agreed in all cases (*n* = 10). Studies were published between 2011 and 2020. A total of 123 patient and 20 control specimens were included in the qualitative synthesis. Control specimens were postmortem human samples without clinical history of neurological disorders. All studies (*n* = 11) provided inclusion criteria for patient selection prior to surgery, whereas few (*n* = 3) reported exclusion criteria. Most studies (*n* = 9) included temporal lobe or hippocampal resection specimens [29, 30, 31, 33, 34, 36–39]. Other studies (*n* = 2) included only extratemporal specimens [32, 35]. Histopathological diagnosis of surgical specimens most often confirmed HS (*n* = 54), mesial temporal sclerosis (MTS, *n* = 6) or FCD (*n* = 58).

**Table 1:** Study design parameters of included studies

Reference	Publication year	Type	Inclusion criteria	Exclusion criteria	Specimen preparation method	Primary endpoint	Measurement of primary endpoint
Garbelli et al. [29]	2011	CS	Drug-resistant TLE w/ HS	-	Fixation in 4% paraformaldehyde for 5 h, post-fixed for 4–5 days	Investigating histopathological substrate of MRI abnormalities in FCD IIIa at 7T	Correlating MRI with histopathology
Garbelli et al. [30]	2012	P	Drug-resistant TLE w/ HS	HS associated with other lesions (tumors, scars, etc.)	Fixation in 4% paraformaldehyde at variable fixation times	Investigating histopathological substrate of MRI temporo-polar blurring in FCD IIIa at 7T	Comparing blurring and non-blurring samples, correlating in vivo 1.5T with ex vivo 7T, histopathological and ultrastructural data
Coras et al. [31]	2014	P	Drug-resistant TLE	-	Fixation in 4% paraformaldehyde for surgical specimens, 4% formalin for postmortem specimens	HS subtype differentiation; HC delineation with 7T MRI; Intrahippocampal connectivity assessment	Qualitative and quantitative multiparametric analysis, comparing MRI with histopathology by two experts
Reeves et al. [32]	2015	P	Adult patients w/ focal epilepsy	-	Fixation in formalin for at least 5 days	Identification of 9.4T qMRI measures and LPA in detection of abnormalities in FCD	Correlating qMRI and LPA with histologically normal and abnormal appearing WM and GM
Modo et al. [33]	2015	CR	Drug-resistant TLE	-	Fixation in 4% paraformaldehyde and post-fixed for 48 h	Best sequence, resolution and 𝛿 for HC delineation with 11.7T MRI; Intrahippocampal connectivity assessment	Qualitative and quantitative multiparametric analysis, comparison MRI with histopathology
Goubran et al. [34]	2015	P	Drug-resistant TLE	-	Fixation overnight in 10% formalin	Validation of in vivo subfield-specific DTI using ex vivo 9.4T scanning	Comparing in vivo 3T/7T with ex vivo 9.4T MRI diffusion parameters
Zucca et al. [35]	2016	P	Neuropathological diagnosis of resected specimens as FCD II	-	Fixation in 4% paraformaldehyde for at least 24 h	Investigating histopathological and ultrastructural substrate of 7T MRI abnormalities in FCD IIa/b	Correlating MRI with histopathological and ultrastructural data
Kwan et al. [36]	2017	R	Drug-resistant TLE, patient age: 16–65	Severe coexisting or terminal systemic disease, dual pathology, unsuitable for MRI evaluation	Fixation overnight in 10% formalin	HC abnormality detection and subfield identification at 9.4T	Qualitative analysis of MRI by two neuroradiologists, comparison of MRI with histopathology
Gillmann et al. [37]	2018	P	Drug-resistant TLE and presurgical indication of HC involvement	-	Fixation in 4% paraformaldehyde for 24 h	HS subtype differentiation at 7T MRI	Qualitative and quantitative multiparametric analysis
Ly et al. [38]	2020	P	Drug-resistant mTLE	-	Fixation in 4% formaldehyde for 48 h	Defining optimal acquisition parameters for dMRI and tractography at 11.7T	Comparing different resolutions, diffusion times, directions, b-values, and sample orientations
Ke et al. [39]	2020	P	Drug-resistant mTLE	Samples < 800 mm^3^; Seizure frequency > 10/month; Pediatric onset of epilepsy	Fixation in 4% formaldehyde, post-fixed for 48 h in surgical specimens, fixation in 10% buffered formalin for 6 weeks in postmortem controls	Determining cell layer specific diffusion and connectivity changes at 11.7T in mTLE	Comparing dMRI epilepsy specimens with controls, tractography

CS = case series. CR = case report. DTI = diffusion tensor imaging. dMRI = diffusion MRI. FCD = focal cortical dysplasia. GM = gray matter. HC = hippocampal. HS = hippocampal sclerosis. LPA = line profile analysis. MRI = magnetic resonance imaging. mTLE = mesial temporal lobe epilepsy. P = prospective. qMRI = quantitative MRI. R = retrospective. T = tesla. TLE = temporal lobe epilepsy. w/ = with. WM = white matter. 𝛿 = diffusion durations. ‘-‘ = not specified

Each study provided a detailed description of the applied MRI protocol, summarized in Table 2. Five studies used a field strength of 7T, three used 9.4T, and three used 11.7T. There was large variation in type of radiofrequency coils between individual studies. Slice thickness varied between 300 and 700 μm, in-plane resolutions between 40 × 40 µm^2^ and 450 × 450 µm^2^. Acquisition times varied from 65 minutes up to 124 hours.

**Table 2:** Ultra-high field MRI parameters of included studies

Reference	T	Scanner	Sequences used	In-plane resolution (µm^2^)	TA
Garbelli et al. [29]	7	BioSpec 70/30 USR, Bruker	T2	73 × 73	-
			T2 (relaxometry)	-	-
Garbelli et al. [30]	7	BioSpec 70/30 USR, Bruker	T2	73 × 73	-
			SE (DTI)	250 × 250	-
Coras et al. [31]	7	BioSpec 70/30 USR; Bruker	2D T2 (morphology)	40 × 40	14 h
			3D T2 (morphology)	156 × 156 × 156	38 h
			EPI (DTI)	109 × 109	15 h
Reeves et al. [32]	9.4	Agilent Technologies	T2 MSE	136 × 136	96 min
			T1 maps MSE	136 × 136	145 min
			T2 maps MSE	136 × 136	102 min
			T2* maps MSGE	136 × 136	75 min
			MTR maps MSGE	136 × 136	65 min
Modo et al. [33]	11.7	Bruker Avance DBX	3D T2 SE	100 × 100	124 h
			3D PGSE (DTI)	100 × 100	
Goubran et al. [34]	9.4	Varian	SE (DTI)	100 × 100	-
			TrueFISP	100 × 100	-
Zucca et al. [35]	7	BioSpec 70/30 USR, Bruker	T2	46 × 46	-
Kwan et al. [36]	9.4	Varian	TrueFISP	-	-
Gillmann et al. [37]	7	ClinScan 70/30, Bruker	3D T2 TSE (morphology)	43 × 43 × 300	207 min
			3D FLASH (relaxometry)	-	5 min
			2D SE (relaxometry)	-	210 min
			2D GRE (relaxometry)	-	14 min
			2D EPI (DTI)	-	8.5 h
Ly et al. [38]	11.7	89 mm Bruker Avance AV3 HD	3D T2 SE	-	-
			3D PGSE (DTI)	100 × 100, 200 × 200, 450 × 450	3 h 30 min – 74 h
Ke et al. [39]	11.7	89 mm Bruker Avance AV3 HD	3D T2 SE	100 × 100	8 h 52 min
			3D PGSE (DTI)	100 × 100	63 h

2D = two-dimensional. 3D = three-dimensional. DTI = diffusion tensor imaging. EPI = echo planar imaging. FLASH = fast low angle shot. GRE = gradient echo. MSE = multi-spin echo. MSGE = multi-slice gradient echo. MTR = magnetization transfer ratio. PGSE = pulsed gradient spin echo. SE = spin echo. T = tesla. T1 = T1 weighted. T2 = T2 weighted. TA = acquisition time. TrueFISP = true fast imaging with steady state precession. TSE = turbo spin echo. ‘-‘ = not specified

Most frequent outcome measures addressed the applicability of UHF MRI sequences to visualize anatomical structures and abnormalities. The majority of included studies (*n* = 9) validated UHF MRI findings by histopathological analysis and/or ultrastructural analysis using electron microscopy. Table 3 summarizes histopathological diagnoses and primary endpoint outcomes.

The level of evidence of included studies ranged from level three to four. The studies were of adequate methodological quality, scoring either good or fair based on the quality assessment tool.

**Table 3:** Histopathological diagnoses and primary endpoint outcome of included studies

Reference	Number of cases	Number of controls	Resection	Histopathological diagnosis	Primary endpoint outcome*
Garbelli et al. [29]	13	-	ATL w/ HCT	FCD IIIa: 9 HS-only: 4	T2 signal intensity is strongly related to myelin and likely to cellular density.
Garbelli et al. [30]	32	-	TL	FCD IIIa: 28 HS-only: 4	Blurring at 1.5T and white matter signal inhomogeneity at 7T is related to degeneration and redistribution of axonal fibers confirmed by histopathology and ultrastructural analysis using electron microscopy.
Coras et al. [31]	18	15	HCT	HS 1: 13 HS 2: 5	High concordance between MRI and histopathology. MD values allowed discrimination between HS 1 and HS 2, structural MRI data and FA did not. All subfields could be visualized in non-HS specimens. Hippocampal fiber tracking could clearly visualize intrahippocampal projections, being distorted in HS specimens.
Reeves et al. [32]	12	1	Frontal, parietal, temporal, occipital cortex resection	FCD IIa : 4 FCD IIb : 3 FCD IIId : 1 HS only: 1 Tumor: 1 (dysembryoblastic neuroepithelial) No lesion found: 2	Significant correlations were found between histopathology and qMRI over all ROI (normal and abnormal). LPA could demonstrate subtle cortical dyslamination in FCD.
Modo et al. [33]	1	-	HCT	MTS: 1	Reliable HC layer distinction required 100 μm resolution; subfield differentiation was possible at 500 μm; tractography afforded a unique systems view of the hippocampus and detected mossy fiber sprouting.
Goubran et al. [34]	5	-	ATL w/ AH	HS: 5	Good agreement between in vivo and ex vivo diffusion parameters MD and FA, observing a slightly lower correlation in CA1 compared to CA4.
Zucca et al. [35]	13	-	Frontal, parietal, temporal, occipital or central cortex resection	FCD IIa: 4 FCD IIb: 9	Inhomogeneous intracortical signal intensity on 7T MRI corresponded to fiber disorganization and abnormal cell aggregates, not consistently detected on 1.5T MRI.
Kwan et al. [36]	5	1	ATL with AH	MTS: 5	High concordance between ex vivo MRI abnormalities and histopathology; HC subfields (CA1-CA4, DG) visually identified in all specimens.
Gillmann et al. [37]	14	-	HCT	HS 1: 10 HS 2: 2 No-HS, gliosis-only: 2	Differentiation between HS subtypes was achieved using quantitative and qualitative ex vivo 7T MRI.
Ly et al. [38]	3	-	ATL w/ HCT	HS: 3	Multiple sets of acquisition parameters capture different anatomical details with dMRI; Tractography revealed intrahippocampal connectivity and reflected histopathological boundaries of hippocampal layers and subfields.
Ke et al. [39]	7	3	HCT	HS: 7	Measurements of volumetric changes within individual hippocampal layers could only be achieved at 100 μm isotropic resolution. Diffusion measurements and streamline density distinguish mTLE from control samples.

*Primary endpoints and outcome measurements are described in Table 1

AH = amygdalahippocampectomy. ATL = anterior temporal lobectomy. CA = cornu ammonis. DG = dentate gyrus. dMRI = diffusion MRI. FA = fractional anisotropy. FCD = focal cortical dysplasia. HC = hippocampal. HCT = hippocampectomy. HS = hippocampal sclerosis. LPA = line profile analysis. MD = mean diffusivity. MRI = magnetic resonance imaging. mTLE = mesial temporal lobe epilepsy. MTS = mesial temporal sclerosis. qMRI = quantitative MRI. ROI = region of interest. T = tesla. TL = temporal lobectomy. w/ = with. ‘-‘ = not specified

### Results of individual studies

Below, we present the most relevant messages of the selected studies, focusing on (I) the ability of ex vivo UHF MRI to identify subtle structural abnormalities, (II) different imaging modalities and post-processing methods, and (III) MRI-histopathology concordance. Ex vivo UHF MRI findings and their histopathological and ultrastructural correlations are described in Table 4. Two articles were not included in this table as they did not present data regarding histopathology [34, 39].

**Table 4:** UHF MRI measures and correlated histopathological and ultrastructural patterns of included studies

Reference	Histopathological diagnosis	Sequence	Ex vivo UHF MRI findings	Associated histopathological findings	Associated ultrastructural findings using electron microscopy
Garbelli et al. [29]	FCD IIIa	T2	• ↑ Intensity IGL (= homogeneous signal between IGL and SGL)	• ↓ Myelin fiber density in IGL	-
			• ↑ Intensity cortical LIV (= absent hypointense LIV)	• ↓ Myelin fiber density in LIV • Altered myelin fiber distribution • ↓ Neuronal density LIV • Blurred borders LIII-IV-V (NeuN)	
			• ↑ Intense line beneath cortical LI	• Abnormal neuronal clustering • ↓ Neuronal density in LII-III • ↑ Cellular concentration outer LII	
			• Flattened line profiles (LPA)	• ↓ Myelin fiber density in IGL • ↓ Neuronal density • Abnormal cortical lamination	
Garbelli et al. [30]	HS + FCD IIIa	T2	• (temporopolar blurring on 1.5T) • Clear GM-WM boundary • Inhomogeneous signal intensity in WM • ↑ Intensity in WM (patchy areas)	• ↓ Number of axons in WM • ↓ Axonal density in WM • ↑ Gliosis in WM • Inhomogeneous WM myelin density	• [axonal degeneration] • ↑ Extra-axonal space [edema] • ↓ Myelinated axons • ↑ Unmyelinated axons • ↑ Variation in axonal size and morphology • ↑ Glial cells, glial processes, and vacuoles • ↓ Circularity axons • ↑ Axon elongation • ↑ Axon circumference • ↓ Area occupied by axons (µm^2^) • Normal myelin sheath thickness • Normal vessel walls
		DTI	• ↓ FA in WM		
		T2	-	• Heterotopic neurons in WM • ↑ Single WM neurons	
Coras et al. [31]	HS	T2	• ↓ Volume HC subfields in PCL • ↑ Signal intensity in PCL	• ↑ Neuronal cell loss in subfields • ↓ Myelin content • ↑ Gliosis • [other histopathological processes]	-
			• Indiscernible GCL • Inhomogeneous signal intensity	• Granular cell dispersion	
			• Loss of distinct HC layers	• Loss of distinct HC layers • Reorganization of fiber bundles	
		EPI (DTI)	• ↑ MD in subfields	-	
			• ↓ FA in subfields	• [shrinkage, fiber alterations]	
			• Disorganized fibers (tractography)	-	
Reeves et al. [32]	FCD**	T1, T2,T2*	• ↑↓ qMRI value	• ↓↑ Myelin density	-
		T1, T2*	• ↓↑ qMRI value	• ↓↑ Neuronal density	
		MTR	• ↓↑ qMRI value	• ↓↑ Myelin density	
	Normal***	T1	• ↑↓ qMRI value in WM	• ↓↑ Myelin density • ↑↓ Neuronal density	
			• ↑↓ qMRI value in GM	• ↑↓ Blood-brain barrier permeability (albumin)	
		T2	• ↑↓ qMRI value in WM	• ↓↑ Myelin density	
			• ↑↓ qMRI value in GM	• ↑↓ Reactive cellular and chronic fibrillary gliosis	
	FCD IIa	T2 T2*	• Altered LPA in cortex	• ↓ Neuronal density • Abnormal neuronal distribution • DN in cortex	
	FCD IIb	T1, T2 T2*	• Flattened line profile in cortex (LPA)	• Abnormal neuronal, myelin and neurofilament cortical layering	
	FCD III	T2	• Abnormal line profiles in cortex (LPA)	• Abnormal neuronal and myelin cortical layering	
			• ↑ Intensity line in superficial and deep cortical layer	• ↑ Neuronal density in superficial and deep layer	
			• ↓ Intensity line in cortex	• ↑ Myelin density line	
Modo et al. [33]	HS	3D PGSE (DTI)	• [aberrant] connectivity between DG and SM/SR	• Neuronal loss in CA3	-
Zucca et al. [35]	FCD IIb	T2	• Patchily distributed inhomogeneous GM signal intensity in cortical mantle • ↑ Intensity IGL (= equal IGL and SGL intensity) • Thickening of GM	• Disorganized intracortical fibers • Disorganized myelinated fibers (radial pattern lost) • ↑ Clustered DN and BC	-
			↑ Intensity (strong) in WM	• Disrupted WM • ↓ Myelinated fibers • ↑ DN and BC • ↓ OLG • Dysmorphic OLG • Reduced cellular density	• ↑ BCs and DN • ↓ Myelinated fibers • Scattered myelinated fibers • Thin/absent myelin sheaths • Dysmorphic OLG • ↑ Microglial cells • ↑ Astroglial cells • Edema
	FCD IIa		• Inhomogeneous GM signal intensity • Blurred GM-WM junction • Cortical disorganization	• DNs in cortical ribbon and WM • Disorganized myelinated fibers (radial pattern lost) • Alteration in cortical architecture	• ↓ Myelinated fibers • Dystrophic axons • ↑ Microglial cells • ↑ Glial cells • ↑ Astroglial cells
Kwan et al. [36]	HS	TrueFISP	Not stated. Samples were classified as abnormal if one of the following features was present: • Atrophy (size reduction) • Architectural irregularities (shape, texture, blurring) • Signal abnormality (hypo-/hyperintensity)	• Severe neuronal loss in CA1 • Moderate neuronal loss in CA2, CA3, CA4 • Diffuse gliosis in HC • Dentate fascia/gyrus depleted and dispersed	-
Gillmann et al. [37]	HS	T2 TSE	• Narrowing of PCL • Hyperintensity in PCL • Broadened and indiscernible DG • Ill-defined boundary DG/ML • ↓ Product of PCL • ↓ Area of PCL	• Neuronal cell loss in subfields (CA1, CA2) of PCL	-
		2D GRE	• ↑ T2*-times		
		2D EPI (DTI)	• ↓ FA • ↑ ADC		
Ly et al. [39]	HS	3D PGSE (DTI)	• ↑ Intensity	• Reactive gliosis • Surgical trauma	-
			• ↓ Volume • ↑ MD	• Neuronal loss	
			• ↑ Streamline density	• [epilepsy induced changes or better tissue quality]	
			• ↑ Streamline variability • ↑ MD, AD, RD variability	• [epilepsy induced changes]	

*Text within square brackets indicate histopathological changes proposed by the authors, but not histopathologically validated

**FCD samples with both normal and abnormal appearing white matter and cortex

***Normal-appearing white matter and cortex in epilepsy samples

AD = axial diffusivity. ADC = apparent diffusion coefficient. BC = balloon cells. CA = cornu ammonis. DG = dentate gyrus. DN = dysmorphic neurons. DTI = diffusion tensor imaging. EPI = echo planar imaging. FA = fractional anisotropy. FCD = focal cortical dysplasia. GCL = granule cell layer. GM = gray matter. GRE = gradient echo. HC = hippocampal. HS = hippocampal sclerosis. IGL = infragranular layer. L = layer. LPA = line profile analysis. MD = mean diffusivity. ML = molecular layer. MRI = magnetic resonance imaging. MTR = magnetization transfer ratio. OLG = oligodendrocytes. PCL = pyramidal cell layer. PGSE = pulsed gradient spin echo. qMRI = quantitative MRI. RD = radial diffusivity. SGL = supragranular layer. SM = stratum moleculare. SR = stratum radiatum. TrueFISP = true fast imaging with steady state precession. TSE = turbo spin echo. UHF = ultra-high field. WM = white matter

Garbelli et al. [29] scanned cortical specimens from thirteen drug-resistant temporal lobe epilepsy (TLE) patients with HS at 7T. Histopathological analysis revealed FCD type IIIa in nine out of thirteen cases, of which five specimens scanned at 7T contained this dysplastic lesion. In specimens with normal cortical layering, a clear boundary between gray and white matter was observed with both MRI and histopathology. Supragranular layers, corresponding to cortical layers I, II, and III, appeared hyperintense when compared to infragranular layers, corresponding to cortical layers V and VI. Furthermore, a hypointense band, corresponding to layer IV, was observed. Line profile analysis (LPA) confirmed this signal intensity dip. In three out of five FCD cases, the intensity difference on MRI between supra- and infragranular layers was absent, with immunocytochemistry showing blurred borders between these layers. In addition, the mid-layer intensity dip was not visible in most cases. Yet, a thin hyperintense longitudinal band was observed just beneath layer I, corresponding to elevated cellular concentrations at the outer border of layer II.

Garbelli et al. [30] scanned nine specimens of the same cohort of the previous study at 7T. Based on presurgical 1.5T MRI evaluation, patients were divided in two groups: four with HS plus ipsilateral temporo-polar gray-white matter blurring (group 1) and five with HS without ipsilateral temporo-polar abnormalities (group 2). Ex vivo T2w images at 7T showed a clear border between grey and white matter in all samples. However, only those of group 1 displayed white matter signal inhomogeneities with patchy areas of hyperintensity, whereas specimens of group 2 showed a homogeneous hypointense signal throughout the white matter. Fractional anisotropy (FA) maps could further differentiate between groups, with lower white matter FA values in group 1. Histopathological analysis revealed inhomogeneous white matter staining in group 1, whereas the same staining was homogenous in group 2. Ultrastructural analysis confirmed a reduction in number of axons and axonal density in samples from group 1 at the level of white matter.

Coras et al. [31] analyzed 33 resected hippocampi at 7T, of which four were postmortem controls from patients without neurologic disorders and 29 specimens from patients with drug-resistant TLE. In non-HS specimens, T2w images revealed a distinct organization of the hippocampal anatomy confirmed by histology. Thus, postmortem UHF MRI permitted differentiation between all seven hippocampal layers and identification of parahippocampal structures. Yet only four layers could be distinguished in HS specimens. T2w images of HS specimens showed several structural alterations, histopathologically correlating to neuronal loss and gliosis. Mean diffusivity (MD) values were significantly higher in HS specimens than in non-HS specimens and were able to discriminate between the HS type 1 and 2. Fiber tractography was performed and revealed a general disorganization of the reconstructed fibers in HS specimens when compared to non-HS specimens.

Reeves et al. [32] scanned cortical resections and underlying white matter from twelve patients with temporal or extratemporal epilepsy and one control sample without history of epilepsy at 9.4T. Application of quantitative MRI (qMRI) allowed detection of discrete abnormalities such as subtle changes in cortical and white matter neuronal numbers and myelin density of normal appearing cortex and white matter. Quantitative analysis of T1, T2, and T2* values at 9.4T revealed a reduction in white matter myelin density, even in a preoperatively 3T MRI-negative FCD type II case. LPA showed abnormal intensity gradients corresponding to abnormal cortical lamination, neuronal populations, and myeloarchitecture. In addition, LPA differed between FCD variants, suggesting the applicability of LPA to distinguish subtypes. A significant correlation between these findings and histopathology was observed.

Modo et al. [33] scanned one resected hippocampus from a drug-resistant TLE patient at 11.7T with subtle indications of mesial temporal sclerosis on a preoperative 3T MRI scan. Apparent diffusion coefficient (ADC) contrast maps provided a detailed view of hippocampal layers and pathology consistent with histological findings. The use of color-coded multiparametric MRI (ADC, FA, T2) highlighted different structural aspects akin to immunohistochemistry. Tractography at 100 μm allowed visualization of intra- and extrahippocampal connections, showing connections between the dentate gyrus and stratum moleculare/radiatum and therefore supporting the mossy fiber hypothesis.

Goubran et al. [34] scanned five resected hippocampi from drug-resistant TLE patients at 9.4T. Diffusion parameters (FA and MD) in cornu ammonis (CA) 1 and CA4 were compared between in vivo and ex vivo scanning, showing good agreement. CA4 showed higher correlations for FA and MD than CA1, possibly due to the curved shape of CA1 compromising gray matter values next to cerebrospinal fluid. Despite good agreement between in vivo and ex vivo diffusion parameters, a shift was observed, proposedly due to fixation and tissue processing effects on the microstructure of the specimens.

Zucca et al. [35] scanned resected specimens from thirteen drug-resistant TLE patients with histopathologically confirmed FCD type IIa (*n* = 4) or IIb (*n* = 9) at 7T. T2w images of FCD type IIb specimens consistently showed inhomogeneity of intracortical signal intensity, histopathologically corresponding to patches of abnormal cells (dysmorphic neurons and balloon cells) and disorganized fibers, alongside white matter hyperintensities, corresponding to dysmyelination in the presence of balloon cells. Quantitative analysis of these images, by calculating mean signal intensity and coefficient of variation (CV, defined as the ratio of the standard deviation to the mean), could distinguish lesional from perilesional tissue. FCD IIa showed less severe histopathological alterations, which is reflected in their less evident MRI alterations.

Kwan et al. [36] scanned five resected HS samples and one non-HS sample from drug-resistant TLE patients at 9.4T. Both neuroradiologists were able to identify all hippocampal subfields in the head and body region on True Fast Imaging with Steady State Precession sequencing. Concordance between UHF ex vivo MR images and histopathology for abnormalities detected in hippocampal subfields and dentate gyrus was high.

Gillmann et al. [37] scanned resected hippocampi from fourteen drug-resistant TLE patients at 7T. T2w images were able to differentiate between HS type 1, type 2, and no-HS, based on visualization of narrowing patterns of the pyramidal cell layers (PCL), concordant with histopathology. Analysis of quantitative parameters showed significant differences between previously stated groups for PCL product (derived by multiplying widths of PCL), PCL area, T2*-relaxation times, FA, and ADC.

Ly et al. [38] scanned three resected hippocampi from drug-resistant TLE patients at 11.7T. MD and FA maps at a spatial resolution of 100 μm provided reliable delineation of hippocampal subfields and lamina, but not at 450 μm. Relatively short diffusion times improved tractography and signal-to-noise contrast in both MD and FA images. Spatial resolution and sample orientation impacted streamline density strongly, but the number of diffusion directions did not. Diffusion and volume measurements were not influenced by sample orientation. High b-values increased contrast on MD images, whereas FA and streamline density showed the opposite trend. Comparison between tractography images and histopathology showed representation of hippocampal anatomy. Connectivity in individual layers as well as between subfields was visualized.

Ke et al. [39] scanned seven resected hippocampi from drug-resistant TLE patients and three postmortem control specimens at 11.7T. Diffusion tensor imaging (DTI) allowed identification of individual hippocampal layers. Diffusivity was higher in hippocampi from TLE patients when compared to controls, and DTI measurements showed more heterogeneity, whilst measurements of controls were more consistent. Directionally encoded color images and tractography on FA maps provided complementary anatomical information.

## Discussion

This review provides a qualitative synthesis of current literature on ex vivo UHF MRI in human tissue samples (*n* = 123 epilepsy, *n* = 20 controls) obtained after epilepsy surgery, covering eleven studies. We focused on (I) the ability of ex vivo UHF MRI to visualize subtle anatomical abnormalities related to epilepsy, (II) different imaging modalities and post-processing methods, and (III) the concordance between histopathology and UHF MRI characteristics.

### Ex vivo MRI

The benefits of ex vivo MRI when compared to in vivo scanning allow us to broaden our understanding of underlying structural aberrances in drug-resistant epilepsy specimens and contribute to the discovery of potential radiological biomarkers, as described in the following paragraphs. However, the disadvantages and limitations of ex vivo scanning should also be noted. Tissue fixation causes shrinkage and dehydration, which can also lead to changes in tissue integrity, possibly resulting in false or misleading findings. The postmortem interval and time in fixative are influential factors and should be kept as short as possible to minimize confounding [40, 41]. Diffusivity of gray and white matter has shown to decrease by 20–40% in a period of six hours before fixation [42]. Another consideration of importance when interpreting ex vivo images, is that fixation can alter MR-parameters. It has been reported that fixatives can decrease relaxation times and water content, and influence diffusion metrics [43–45]. While these alterations influence MRI sequence parameters of all contrasts, they are especially impactful and challenging for ex vivo diffusion MRI, requiring the implementation of various mitigation strategies [40]. Addressing reduced diffusivity and lower T2 values in fixed ex vivo tissue, which significantly diminish the signal-to-noise ratio and diffusion contrast, begins with tissue processing methods in attempt to counteract these effects (e.g. by rehydration). Additionally, embedding the tissue in a susceptibility-matching, proton-free medium (e.g. Fluorinert or Fomblin) helps prevent excessive magnetic susceptibility gradients at tissue boundaries. Of greater importance is the requirement for much higher diffusion weighting due to the reduced diffusivity, and the requirement for much shorter MRI readouts to avoid image blurring due to the reduced T2 values. Achieving higher diffusion weighting and shorter readouts is more feasible with advanced gradient systems in small and medium-bore preclinical MR systems, which were used in the tractography studies included in this review. However, these modifications to MRI parameters, and MRI protocol changes to adjust for them, can complicate the translation of ex vivo findings to in vivo setting and their pathobiological interpretation. These factors should always be kept in consideration during their interpretation.

### Ex vivo UHF MRI findings

#### Hippocampal sclerosis

The following ex vivo MR features were characteristic of HS, enabling differentiation from non-HS or non-epilepsy controls: qualitative hippocampal volume loss, T2 signal hyperintensity changes, increased T2* relaxation times, and architectural alterations in hippocampal subfields [31, 36, 37, 39]. These abnormalities have been previously described in conventional field in vivo MRI studies [46–48].

The hippocampus is formed by the infoldings of the dentate gyrus, cornu ammonis, and subiculum, of which the latter is continuous with the six-layered neocortex. The three cortical layers of the hippocampus, collectively known as the archicortex, consist of a superficial molecular layer and a deep polymorphic layer. The middle layer typically contains pyramidal cells, except in the dentate gyrus, where it consists of granular cells. The cornu ammonis can be further subdivided into regions CA1, CA2, CA3, and CA4. HS classification according to the ILAE differentiates between three subtypes based on histopathological patterns of pyramidal cell loss and gliosis in the aforementioned hippocampal subfields [28]. Reliable identification of these cell layers and subfields is relevant, as clinical presentation and prognosis differs between HS subtypes [49]. In the human brain, the thickness of hippocampal sublayers varies between 100 and 500 μm, requiring extremely small voxel volumes to clearly visualize intrahippocampal structures [50]. Intrahippocampal subfields were clearly discernable at spatial resolutions of 500 μm, and even all seven individual cell layers and borders in non-HS hippocampi could be distinguished on T2w images, but only at a minimum of 100 μm [33, 38, 39]. Corresponding acquisition times to achieve this resolution of 100 μm ranged from 8 to 124 hours. Comparable studies visualizing hippocampal subfields and layers have reported similar spatial resolutions and acquisition times [21, 51, 52]. Hippocampal subfields have been depicted in in vivo UHF studies, but reliable discrimination between individual hippocampal layers remains challenging, as spatial resolutions could only reach 200 μm at best [53, 54].

Three of the included studies assessed whether UHF MRI could discriminate between ILAE HS type 1 and 2 [31, 37, 39]. One study was able to differentiate between subtypes by qualitative analysis of T2w images, based on patterns of narrowing of the PCL, as HS type 1 showed narrowing of the PCL in the whole CA1 area and HS type 2 only the upper half in direction to CA2 [37]. Quantitative analysis showed a significant difference between the parameter ‘product’ of HS type 1 and 2 [37]. Likewise, differences in MD values enabled reliable discrimination between both subtypes [31]. In clinical settings, conventional MRI has not been able to discriminate between subtypes since visualization of hippocampal structures is limited by resolution and contrast [37].

#### Focal cortical dysplasia

Ex vivo UHF MRI of epilepsy specimens was used to study the intracortical architecture of both normal and pathological cortical regions. Three of the included studies investigated areas with histopathological normal-appearing cortex [29, 32, 35]. The neocortex is structured into six distinct layers, numbered I through VI, each composed of unique neuron types and connections. Layer IV, referred to as the granular layer because of its characteristic small, densely packed granule cells, acts as the boundary between the supra- (I-III) and infragranular layers (V and VI). Differentiation between supra- and infragranular layers was achieved based on signal intensities visualized with T2w images, reporting similar intracortical intensity profiles as previous studies [55–57]. However, despite achieving high resolution images of the cortex at 7T and 9.4T, included studies were still unable to separate all six cortical layers individually. With the UHF T2w sequences that were used, myelin contrast was still insufficient, which could be a region-specific limitation.

Four studies focused on the depiction of abnormalities related to FCD with ex vivo UHF MRI [29, 30, 32, 35]. T2w and T2*w sequences provided the most valuable contrast for visualizing structures, in comparison with T1w sequences and magnetization transfer ratio maps [32]. These findings are in line with other studies, considering T2w or T2*w sequences most suitable for ex vivo examination of the cortical architecture [58, 59].

Two studies examined FCD type II specimens, where UHF MRI could detect patches of inhomogeneous intracortical signal intensities on T2w images, corresponding to abnormal cells and fiber disorganization in histological slices [32, 35]. This inhomogeneity seemed exclusive for FCD type II, as type III cases were characterized by a homogeneous signal throughout the cortex [29]. To date, intracortical signal inhomogeneity has not been described as a typical feature of FCD type II, thereby presenting itself as a possible novel radiological biomarker for this subtype [60].

Three studies examined FCD type III specimens [29, 30, 32]. FCD type III lesions consist of cortical architectural aberrations in close association with primary lesions, like HS (type IIIa), acquired during early life [61]. Detected features in FCD IIIa cases included: signal homogeneity between supra- and infragranular layers, absence of a mid-cortical hypointense signal, a superficial cortical hyperintense signal, and white matter signal inhomogeneity. In addition, white matter abnormalities in FCD IIIa were associated with reduced FA values [30]. Accordingly, previous studies have shown decreased FA values corresponding to white matter abnormalities in the temporal lobe, external capsule, corpus callosum, and cingulum in patients with TLE and HS [62–65]. The underlying cause of this FA reduction is not yet conclusive as reduced myelin content, axonal loss, altered myeloarchitecture, and increased extra-axonal space and axonal circumference have all been postulated, but not yet confirmed by histopathology [62, 65, 66]. In correspondence, Garbelli et al. confirmed most of these proposed abnormalities by performing ultrastructural analysis in white matter regions with reduced FA values [30].

### Post-processing techniques

#### Post-processing techniques in HS

Several studies investigated DTI parameters in HS and compared results with either non-HS TLE samples or postmortem controls. The findings suggest that increased MD, ADC, axial diffusivity, and radial diffusivity are characteristics of HS [31, 37, 39]. These results are in concordance with prior studies utilizing dMRI in patients with HS-associated epilepsy [67–69]. In contrast, data on FA values appeared inconsistent between studies. One study showed significant lower FA values in HS compared to non-HS [35], whereas two other studies found FA data to be uninformative [31, 39]. Significantly lower FA values have previously been reported in patients with HS, being linked to reduced myelin density [68–70]. Microstructural changes, such as neuronal or axonal loss, are thought to result in lower anisotropy and increased diffusivity [71]. However, diffusion measurements are inherently non-specific, as changes in the values mentioned above are influenced by different types of microstructural processes [70, 72]. Regardless, considering that microstructural changes in epilepsy may precede or occur alongside alterations at a macrostructural level, implies an important role for diffusion parameters in providing complementary diagnostic information in HS [73, 74].

The quantitative parameters area and product, alongside volumetric analysis in specific cell layers allowed reliable assessment of hippocampal subfield volume reductions in HS [31, 36, 39]. Furthermore, LPA methods appeared useful in the detection and corroboration of hippocampal layer disruption [31].

To examine connectivity in hippocampi, tractography was applied in four included studies [31, 33, 38, 39]. Because UHF MRI resulted in much smaller voxel sizes, the detrimental impact of partial volume effects on tractography was minimalized [75]. Compared with non-HS specimens, sclerotic hippocampi clearly showed disorganization of fiber networks, suggestive of fiber rearrangement during sclerotic processes. Potential aberrant connections between the granular cell layer of the dentate gyrus and the stratum lacunosum-moleculare in CA3 were determined in HS samples, which have been widely hypothesized as mossy fiber sprouting and are believed to underlie aberrant excitatory circuits [76–78]. However, little is known about the mossy fiber hypothesis. It is unclear whether these aberrant connections are necessary or even sufficient to cause drug-resistant epilepsy and might even be present in non-epilepsy specimens. In accordance with the latter, one study showed connectivity between the granular cell layer and stratum lacunosum-moleculare regions in non-epileptic postmortem control samples [39]. The presence of glutamatergic axons between these regions in healthy controls would question the hypothesis. Alternatively, this finding could be explained by imprecise generation of tracts, which may have resulted in falsely observed connectivity between regions. All four included studies relied on a fiber reconstruction model known to present strong limitations, as DTI-based tractography cannot render multiple fibers within a single voxel (“crossing-fiber” problem) [72]. This is a major flaw in tissues with complex circuitry such as the hippocampus. It would be interesting to replicate results with more sophisticated multi-shell hybrid diffusion imaging acquisition schemes, enabling reconstruction of more complex fiber configurations, such as crossing, splitting, and kissing fibers [72].

Diffusion-encoded color maps provided complementary information in hippocampal specimens, aiding in delineation of different hippocampal regions [38, 39]. Color-coded multiparametric MRI highlighted histological aspects of tissue and further refined boundaries between single layers [33, 39].

#### Post-processing techniques in FCD

Despite enhanced visualization of lesions, very discrete abnormalities in some FCD cases still escaped detection. Several post-processing methods, such as LPA, CV, and qMRI, demonstrated the ability to detect these aberrances, suggesting their applicability in patients with MRI-negative epilepsy.

LPA reflected abnormalities in myeloarchitecture, cytoarchitecture, and cortical lamination [29, 32]. These results suggest that LPA may facilitate the detection of dyslamination patterns in FCD and could potentially play a role in the differentiation between subtypes.

Another approach, explored by one study, was based on calculation of the CV within cortical lesional and perilesional regions on T2w images [35]. Interestingly, calculating the CV enabled the distinction of cortical lesional and perilesional areas, even in a preoperative 1.5T MRI-negative patient and ex vivo 7T MR images without abnormalities. These findings demonstrate the potential of CV calculation to enhance the detection of occult MRI lesions, together with border delineation of the lesion. Accurate border identification is challenging, as adjacent perilesional areas often contain discrete abnormalities not readily visible on MRI [79].

One study applied qMRI, where maps of physical or chemical variables are measured in physical units and can be compared between tissue regions and among subjects [80]. Subtle differences were found in myelination and neuronal density between normal-appearing cortex and white matter of specimens derived from epilepsy patients [34].

### Histopathology and UHF MRI concordance

Hippocampal cell layers and subfields observed with ex vivo UHF MRI were reported as highly concordant to histopathology [31, 36], and qualitative assessment of MRI alterations in different hippocampal subfields (CA1-CA4) were confirmed by histopathological abnormalities [31, 33, 36, 37]. In general, hippocampal volume loss is linked to reduced neuronal density, whereas T2 signal hyperintensity is believed to indicate gliosis [81–84]. However, in one included study, quantitative analysis of T2 signal and subfield volumes did not significantly correlate to neuronal cell density, gliosis, or myelin density [31]. These results may suggest that T2 signal and volume changes in HS are caused by a combination of additional pathological processes (e.g. fiber disorganization, myelinated fiber alterations, water content and extra-axonal space changes).

All included studies on FCD analyzed correlative histopathology, including two studies performing ultrastructural analysis using electron microscopy [29, 30, 32, 35]. Visually detected aberrances on 7T and 9.4T MRI consistently correlated to structural alterations in corresponding areas, whilst the severity of histopathological features was reflected by the magnitude of MRI alterations. FCD type IIa showed less severe histopathological abnormalities compared to type IIb cases [35]. Correspondingly, MRI features of type IIa were less evident and more difficult to detect, even at UHF MRI [32, 35]. Especially the presence of balloon cells in FCD type IIb seems to correspond with signal alterations on MRI. A similar trend has previously been reported in conventional in vivo MRI, as up to 49% of patients with type IIa and only 10% of those with type IIb have shown normal or diagnostically unspecific 3T MRI features [85]. Additionally, T2 signal intensity changes were mainly related to myelinated fiber and neuronal cell density, consistent with previous observations [55, 56, 86]. White matter hyperintensity was linked to several ultrastructural findings, such as demyelination and the presence of dysmorphic cells. Hypothetically, a combination of pathological changes as described above operate synergistically to alter MRI signal in FCD.

### Limitations

Substantial heterogeneity in MRI protocols limits the comparability of included studies. Sequences, magnetic field strengths, scanning times, acquisition parameters, and post-processing methods differed between studies. Each magnetic field strength requires a unique approach regarding acquisition, increasing dissimilarity between studies [87]. Despite the growing number of UHF MRI scanners in research and clinical settings, experience in their assessment is still limited when compared to conventional MRI. It is therefore of utmost importance for studies to describe their used MRI protocol in detail. The majority of included studies did not disclose how images were assessed and interpreted. Radiological expertise, interrater agreement, or blinding to histopathological diagnosis were frequently not mentioned, potentially affecting the validity and reliability of outcomes.

This systematic review is further limited due to a sparce number of studies available assessing the use of ex vivo UHF MRI in human epilepsy specimens. Of the included studies, sample sizes were relatively small, together with an uneven distribution of HS and FCD subtypes. As a result, data regarding specimens of FCD type I were not available. Research on FCD type I consequently remains limited when compared to type II and III. Reports on incidence, clinical presentation, imaging features, and specific molecular or genetic biomarkers are either missing or inconclusive, resulting in challenging treatment strategies [88, 89]. Likewise, few non-pathological control specimens were included. This limits our ability to differentiate pathological changes from normal anatomical variations, which is crucial for accurate diagnosis and treatment strategies like surgery planning.

Finally, only few of the reviewed articles provided clinical information of patients from whom the surgical specimens were obtained. As a result, important clinical variables, such as age, disease duration or severity – which could influence pathological processes – could not be assessed as potential confounders in this systematic review. In addition, exploring correlations between clinical data, particularly postoperative outcome, and ex vivo MRI findings could yield valuable insights into the prognostic value of these imaging findings.

### Future perspectives

UHF MRI has proven to be a sophisticated tool in the detection and delineation of epileptogenic lesions and in broadening our understanding of the pathophysiology of epilepsy. Translation of ex vivo findings to in vivo imaging in clinical settings could therefore further enhance the ability of in vivo UHF MRI in not only diagnosing epilepsy, but also in guiding surgical treatment. By visualizing lesions and perilesional regions in greater detail, and transferring these images to the neuronavigation, UHF MRI could result in a more precise and complete resection.

Post-processing analysis techniques have shown to be a promising method for improving diagnostic gain and should be further explored in UHF MRI [90–93]. Relatively new approaches and advances in MRI techniques, such as DTI and tractography, are becoming increasingly important in the radiological diagnosis of patients with epilepsy [94]. Another modality, MR spectroscopy, assesses metabolite levels in the brain and has shown to provide complementary information, especially in MRI-negative epilepsy patients [95–97]. Ex vivo UHF MR spectroscopy assessment could provide metabolic information with higher spectral resolution and sensitivity, improving characterization of abnormal metabolic patterns in lesional areas. Correlating MR spectroscopy metabolites to immunohistochemistry may detect surrogate biomarkers of epileptogenic tissue [98]. To elevate the profitability of UHF MRI, we emphasize the use of these modalities in specialized centers as a potential tool to investigate microstructural changes.

Although ex vivo UHF MRI research has primarily centered around FCD (type II and III) and HS (type 1 and 2) subtypes, a significant gap in studies exploring its potential for other epileptogenic lesions through MRI-histopathology correlations remains. Future ex vivo UHF MRI studies should aim at inclusion of larger numbers of epilepsy specimens to increase the power of their findings, specifically focusing on the involvement of FCD type I, HS type 3, no-HS gliosis only hippocampi, and other epileptogenic lesions, while correlating findings with non-pathological control specimens. MRI-histopathology correlation studies could assist in bridging the gap of knowledge on these epileptogenic lesions, equivalent to what it has achieved for other (sub)types.

## Conclusion

This systematic review identified eleven studies utilizing ex vivo UHF MRI at 7T, 9.4T, and 11.7T as an imaging method for the assessment of surgically resected specimens from patients with drug-resistant epilepsy. Ex vivo UHF MRI has clear value to visualize hippocampal and cortical structures in histopathological detail, enabling detection of subtle, potentially epileptogenic, abnormalities. Advanced post-processing methods allow extraction of valuable additional information, further increasing the potential of UHF MRI. The high concordance between ex vivo MR images and histopathology demonstrates the reliable reflection of tissue geometry by MRI, and further enhances our knowledge about potential radiological biomarkers.

Future studies with larger sample sizes, inclusion of all subtypes of epileptogenic lesions, uniform imaging protocols, innovative sequences, and novel post-processing methods, may enable new methods to be translated into clinical applications, eventually improving our understanding of epilepsy, diagnosis, surgical decision making, and postsurgical outcomes.

## Electronic supplementary material

Below is the link to the electronic supplementary material.

Supplementary Material 1

## Data Availability

All data analyzed during this systematic review are included in the published article and its supplementary material files. No new primary data were collected for this review. Any additional information is available from the corresponding author upon reasonable request.
