# Supplementary material for: Ex vivo ultra-high field magnetic resonance imaging of human epileptogenic specimens from primarily the temporal lobe: A systematic review
Source: Neuroradiology. 2025 Mar 8;67(4):875–93. doi: 10.1007/s00234-024-03474-0 (PMC12041060; doi:10.1007/s00234-024-03474-0)
Supplement: Supplementary file 1 — Supplementary Material 1 [file 234_2024_3474_MOESM1_ESM.docx]

**Supplemental information**

**1. Full search strategies**

PubMed (Medline): ("Epilepsy"[MeSH] OR epilep*[TW] OR seizur*[TW]) AND ("Magnetic Resonance Imaging"[Mesh] OR "magnetic resonance imaging"[TW] OR ("magnetic"[TW] AND "resonance"[TW] AND "imaging"[TW]) OR "mri"[TW] OR "mr"[TW]) AND ("7T"[TW] OR "7 t"[TW] OR "7Tesla"[TW] OR "7 tesla"[TW] OR "7.0T"[TW] OR "7.0 T"[TW] OR "7.0 Tesla"[TW] OR "7.0Tesla"[TW] OR ("seven"[TW] AND "Tesla"[TW]) OR "9.4T"[TW] OR "9.4 t"[TW] OR "9.4Tesla"[TW] OR "9 4 tesla"[TW] OR ("ultra"[TW] AND "high"[TW] AND "field"[TW]) OR "UHF"[TW]) Sort by: Publication Date. 241 results.

Embase (Ovid): (exp epilepsy/ or (epilep* or seizur*).ab,ti,kw.) and (magnetic resonance imaging/ or (magnetic resonance imaging or (magnetic and resonance and imaging) or mri or mr).ab,ti,kw.) and ("7T" or "7 T" or "7Tesla" or "7 Tesla" or "7.0T" or "7.0 T" or "7.0Tesla" or "7.0 Tesla" or (seven and tesla) or "9.4t" or "9.4Tesla" or "9.4 Tesla" or (ultra and high and field) or uhf).ab,ti,kw. 372 results.

Cochrane library (Wiley): (epilep* or seizur*):ti,ab,kw AND (“magnetic resonance imaging” or (magnetic and resonance and imaging) or mri or mr):ti,ab,kw AND (7T or "7 T" or 7Tesla or "7 Tesla" or "7.0T" or "7.0 T" or "7.0Tesla" or "7.0 Tesla" or (seven and tesla) or "9.4t" or "9.4 t" or "9.4Tesla" or "9.4 Tesla" or (ultra and high and field) or uhf):ti,ab,kw. 8 results.

Web of Science Core collection: TS=(epilep* or seizur*) AND TS=(“magnetic resonance imaging” or (magnetic and resonance and imaging) or mri or mr) AND TS=(7T or "7 T" or 7Tesla or "7 Tesla" or "7.0T" or "7.0 T" or "7.0Tesla" or "7.0 Tesla" or (seven and tesla) or "9.4t" or "9.4 t" or "9.4Tesla" or "9.4 Tesla" or (ultra and high and field) or uhf). Indexes=SCI-EXPANDED, SSCI, A&HCI, CPCI-S, CPCI-SSH, ESI. Timespan=All years. 302 results.

ClinicalTrials.gov: ((MRI) OR (magnetic resonance imaging)) AND ((UHF) OR (ultra-high field) OR (7T) OR (9.4T) OR (7 Tesla) OR (9.4 Tesla)) | Epilepsy. 12 results.

**2. Ultra-high field MRI parameters of included studies**

| Authors | T | Scanner | Sequences used | TR (ms) | TE (ms) | NA | ST (μm) | In-plane resolution (μm) | Matrix  (mm) | FOV (mm) | TA | 𝛿 (ms) | t_D_ (ms) | Δ (ms) | b-values (s/mm^2^) | DD |
| --- | --- | --- | --- | --- | --- | --- | --- | --- | --- | --- | --- | --- | --- | --- | --- | --- |
| Garbelli et al  2011 | 7 | BioSpec 70/30 USR, Bruker | T2 | 4300 | 50 | 36 | 700 | 73x73 | 256x256 | 32x32 | - | - | - | - | - | - |
|  |  |  | T2 (relaxometry) | 5000 | 13, 26, 39, 52, … 208 | 4 | 700 | - | 256x256 | 44x44 | - | - | - | - | - | - |
| Garbelli et al  2012 | 7 | BioSpec 70/30 USR, Bruker | T2 | 4300 | 50 | 36 | 700 | 73x73 | 256x256 | 32x32 | - | - | - | - | - | - |
|  |  |  | SE  (DTI) | 5000 | 40 | 1 | 700 | 250x250 | 128x128 | 32x32 | - | - | - | - | - | 6 |
| Coras et al  2014 | 7 | BioSpec 70/30 USR; Bruker | 2D T2  (morphology) | 5000 | 60 | 36 | 600 | 40x40 | - | 28x28 | 14h | - | - | - | - | - |
|  |  |  | 3D T2  (morphology) | 6000 | 60 | 14 | 600 | 156x156x156 | - | 28x28x28 | 38h | - | - | - | -- | - |
|  |  |  | EPI  (DTI) | 9000 | 57 | 36 | 700 | 109x109 | - | 28x28 | 15h | - | - | - | - | 126 |
| Modo et al  2015 | 11.7 | Bruker Avance DBX | 3D T2 SE | 4000 | 10 | 1 | - | 100x100 | 256x128x128 | 25.6x12.8x12.8 | 124h | - | - | - | - | - |
|  |  |  | 3D PGSE  (DTI) | 1100 | 28 | 1 | - | 100x100 | 256x128x128 | 25.6x12.8x12.8 |  | 2.5, 4, 6.5 | 12.8, 13.6, 14.16 | 15 | 400 | 6 |
| Reeves et al  2015 | 9.4 | Agilent Technologies | T2  MSE | 4500 | 55 | - | 500 | 136x136 | - | 35x35 | 96 min | - | - | - | - | - |
|  |  |  | T1 maps MSE | 300/500/800/1300/2000/4000 | 10 | - | 500 | 136x136 | - | 35x35 | 145 min | - | - | - | - | - |
|  |  |  | T2 maps  MSE | 2000 | 12.6/20/35/60 | - | 500 | 136x136 | - | 35x35 | 102 min | - | - | - | - | - |
|  |  |  | T2* maps  MSGE | 290 | 5.2/7/9/11/13/20 | - | 500 | 136x136 | - | 35x35 | 75 min | - | - | - | - | - |
|  |  |  | MTR maps  MSGE | 260 | 5 | - | 500 | 136x136 | - | 35x35 | 65 min | - | - | - | - | - |
| Goubran et al  2015 | 9.4 | Varian | SE  (DTI) | 7.6 | 3.8 | - | 400 | 100x100 | 380x256 | 38x25.6 | - | - | - | - | - | - |
|  |  |  | TrueFISP | 7.6 | 3.8 | - | - | 100x100 | - | 38x25.6x19.2 | - | - | - | - | - | - |
| Zucca et al  2016 | 7 | BioSpec 70/30 USR, Bruker | T2 | 6000 | 60 | 40 | 700 | 46x46 | 700x700 | 32x32 | - | - | - | - | - | - |
| Kwan et al  2017 | 9.4 | Varian | TrueFISP | 7.6 | 3.8 | - | - | - | - | 38x25.6x19.2 | - | - | - | - | - | - |
| Gillmann et al  2018 | 7 | ClinScan 70/30, Bruker | 3D T2 TSE  (morphology) | 8520 | 95 | 4 | 300 | 43x43x300 | 704x704 | 30x30 | 207 min | - | - | - | - | - |
|  |  |  | 3D FLASH  (relaxometry) | 20 | 2.5 | 3 | 300 | - | 576x576 | 30x30x30 | 5 min | - | - | - | - | - |
|  |  |  | 2D SE  (relaxometry) | 7210 | 10.3-51.1 | 3 | 300 | - | 576x576 | 30x30 | 210 min | - | - | - | - | - |
|  |  |  | 2D GRE  (relaxometry) | 100 | 4-40 | 3 | 300 | - | 576x576 | 30x30 | 14 min | - | - | - | - | - |
|  |  |  | 2D EPI  (DTI) | 8000 | 50 | 3 | 300 | - | 100x100 | 30x30 | 8.5h | - | - | - | 0, 200, 400, 600, 800, 1000 | 12, 48, 256 |
| Ly et al  2020 | 11.7 | 89 mm Bruker Avance AV3 HD | 3D PGSE (DTI) | 1100 | 25, 34.9, 109.9 | 1 | - | 100x100, 200x200, 450x450 | 256x128x128 | 25.6x12.8×12.8 | 3.5 - 74h (total: 723h) | 4 | 13.6, 23.7, 98.7 | 15, 25, 100 | 1000, 4000, 10000 | - |
|  |  |  | 3D T2 SE | - | - | - | - | 100x100 | - | - | - | - | - | - | - | - |
| Ke et al  2020 | 11.7 | 89 mm Bruker  Avance AV3 HD | 3D T2 SE | 4000 | 10 | 1 | - | 100x100 | 256×128×128 | 25.6×12.8x12.8 | 8h 52min | - | - | - | - | - |
|  |  |  | 3D PGSE (DTI) | 1100 | 25 | - | - | 100x100 | - |  | 63h | 4 | 13.6 | 15 | 4000 | 12 |

DTI = diffusion tensor imaging. DD = diffusion directions. EPI = echo planar imaging. FLASH = fast low angle shot. FOV = field of view. GRE = gradient echo. MSE = multi-spin echo. MSGE = multi-slice gradient echo. MTR = magnetization transfer ratio. NA = number of averages. PGSE = pulsed gradient spin echo. SE = spin echo. ST = slice thickness. t_D_ = diffusion time. T1 = T1 weighted. T2 = T2 weighted. T = tesla. TA = acquisition time. TE = echo time. T2 = T2 weighted. TR = repetition time. TrueFisp = True fast imaging with steady state precession. TSE = turbo spin echo. T1 = T1 weighted. 2D = two-dimensional. 3D = three-dimensional. 𝛿 = diffusion duration. Δ = diffusion spacing.
